# Stigma in Leprosy—Are We Doing Enough?

**DOI:** 10.4269/ajtmh.24-0220

**Published:** 2024-08-27

**Authors:** Harish Kumar Sagar, Harpreet Singh Pawar

**Affiliations:** Clinical Division, ICMR-National JALMA Institute for Leprosy & Other Mycobacterial Diseases, Agra, India

“Where is this unpleasant smell coming from?” I asked my colleague on reaching the outpatient department on a chilly morning of the harsh north Indian winter. “Again, leprosy stigma has affected the poor harder,” replied my colleague while pointing at a distraught young man lying down in the waiting area. As I approached him, the stench emanating from the patient became unbearable and was repelling other individuals. With no one accompanying him, the sorrowful expression on his face suggested a desire for more than mere medical management. As I asked about his illness, tears rolled down his cheeks. After a brief pause, he shared his ordeal, and the immense gravity of the situation weighed heavily on me as I sought to understand the severity of the condition. He was a 28-year-old, unmarried man who used to earn his livelihood as an artisan skilled at crafting intricate wooden toys for children. On a fateful day, he felt feverish and noted extensive eruptions over his trunk and legs. A local practitioner did not suspect the reactional stage of Hansen’s disease but prescribed some injections and oral tablets that made him felt better for a few days. His illness rendered him unable to manage work, and his financial situation deteriorated rapidly. Furthermore, his family ostracized him. It appeared he had not taken a bath in days. He was also malnourished and dehydrated. He was febrile with widespread tender nodular skin lesions, purulent ulcers on his trunk and soles of his feet, and had numerous healed scars bearing witness to his silent battle. He politely requested admission to the hospital because there was no one to take care of him at home. After a thorough examination, we diagnosed him with a case of erythema necroticum ulcerans, a rare but severe vasculonecrotic manifestation of lepromatous leprosy.

He was admitted to the ward. It seemed to me that he felt more relieved upon admission, which ensured timely food and care. On reaching the ward, he could not resist asking for lunch. Having gone without food for more than 2 days, his hunger was insatiable. At ward rounds the following day, he was found to be meticulously organized. He appeared to have some relief from his symptoms compared with his initial presentation. Further management was discussed with colleagues. His condition improved significantly over the next 2 weeks, but he continued to experience new eruptions and neuropathic pain, consistent with the reactional phase. After almost 3 weeks and improved overall physical status, he suddenly noticed weakness of the ring and little finger of his dominant hand and was unable to grasp anything properly. “Sir! Something has happened to my right hand since last night,” he told me worriedly with folded hands during my routine inpatient rounds. On examination, I reassured him that this would resolve in a matter of days and not to worry. It was ulnar neuritis. I started high-dose steroids, which brought some relief. Despite all the efforts of medical management and physiotherapy, some paresis persisted, which progressed to leprosy-associated claw hand deformity, affecting his dexterity.

As ambulatory patients were routinely sent to barber shops for haircuts, I recommended that he go for a haircut at the nearby market. The next day, he told me that the barber turned him away because he was a leprosy patient and it would affect his business. He mentioned it casually, as if habituated to such retorts from others. However, I found it quite unsettling to hear, and it prompted me to think about the deep-rooted societal stigma of leprosy. With more than a month of hospital care, he made remarkable progress, but his right-hand partial claw deformity persisted. His ulcers had healed. His reaction had subsided, and his neuropathic pain was well managed. We planned for hospital discharge. He expressed concerns that his hand deformity might worsen social stigma within his community and potentially hinder his ability to earn a living. He was counseled that reconstructive surgery was required to correct claw hand and that this could be done only after clear demonstration that recurring reactions had subsided. He was also counseled that the hypoesthesia would persist in the affected hand, and he needed to be extra diligent in self-care of this hand. Having regained better physical health, he seemed ready to resume his work with renewed confidence. However, before discharge, I also thought it would be pertinent to talk to his family about the disease, alleviate their doubts, and generate family support. After initial hesitation, his elder brother and father agreed to visit our center. They raised several queries about the cause, prognosis, impact of his illness on other family members, deformity, and his marriage prospects. With all queries answered to their satisfaction, they readily took him home. The role of family education about the condition and counseling for patients with limited family support cannot be emphasized enough. Family-level interventions fostered understanding and empathy and promoted a more accepting environment. Finally, we discharged him, confident in his resilience and wishing him good health—both physically and in his community interactions.

While attending to the patients every day, a thought always stays with me that stigmatized leprosy patients bearing the burden of physical deformity should be at the heart of a holistic approach to care, and these cases represent only the tip of the iceberg.

